# The resilience of ethical assets against different uncertainty shocks

**DOI:** 10.1016/j.heliyon.2024.e40980

**Published:** 2024-12-06

**Authors:** Md Bokhtiar Hasan, M. Kabir Hassan, Mamunur Rashid, Tanzila Akter, Humaira Tahsin Rafia

**Affiliations:** a Department of Finance and Banking, Islamic University, Kushtia, 7003, Bangladesh; b Department of Economics and Finance, University of New Orleans, New Orleans, LA, 70148, United States; c Christ Church Business School, Canterbury Christ Church University, United Kingdom

**Keywords:** Ethical assets, Uncertainties, Resilience, Quantile coherence, Wavelet coherence, Safe haven

## Abstract

The focus of this research is to examine the safe-haven properties of seven ethical and conventional asset classes using two sophisticated techniques: quantile coherence and Wavelet coherence. We analyze data ranging from October 3, 2011, to September 30, 2021, that encapsulates several global risk events. The results exhibit either positive or neutral associations between most assets and the Geopolitical Risk (GPR), indicating their safe haven capabilities against the GPR shocks. Notably, the coherence observed between the Economic Policy Uncertainty (EPU) and these assets reveals a positive correlation during bearish markets (monthly frequency) and normal and bullish markets (weekly frequency). Furthermore, only the S&P Green Bond (SPGRNB) as well as S&P Global Clean Energy (SPCE) indices demonstrate protective attributes against EPU shocks during COVID-19. Conversely, market volatility (VIX) was found to negatively impact all asset classes except SPGRNB, which indicates the non-idiosyncratic nature of VIX shocks. Consequently, investors and fund managers operating in ethical markets may consider optimizing their portfolios to shield their wealth amidst instances of extreme and enduring shocks.

## Highlights

• We examine the safe-haven properties of ethical and conventional assets.
• Novel quantile coherence and Wavelet coherence techniques are employed.
• Most asset classes provide a safe haven against geopolitical risk.
• Most assets also offer a safe haven against EPU, depending on frequencies and times.
• No asset is protective against market volatility (VIX).

## 1 Introduction

Investors opt for safe-haven assets to safeguard the value of their assets during economic, policy, and other naturally occurring uncertainties. Active search for safer alternatives and extreme fear of losing value during uncertain times negatively impact investor risk aversion and increase market trading, risk premium, and overall uncertainty [[1], [2], [3]]. Hence, switching assets and rebalancing portfolios due to uncertainties find their roots in rational expectations and sentimental exuberance. Rational expectations to maximize utility, investors used to shift to conventional safe-haven assets that included precious metals, different commodities, and even currencies. Desperate investors driven by fear may inadvertently choose riskier assets, exacerbating volatility and portfolio rebalancing costs [4,5].

In the recent years, green and ethical assets have experienced significant growth. According to Bloomberg, the worldwide ESG (Environment, Social, & Governance) asset market size is projected to reach US $53 trillion by2025.^1^ PwC estimates institutional investors’ interest growth for ESG investment to soar at 84 % to reach $33.9 trillion by2026.^2^ Aside from social responsibility and growing market size, investments in ethical assets have grown over the years as they have outperformed other asset classes from different dimensions. Investors pay a premium for GBs [6,7]. Green bonds may offer an efficient hedge against short-term market uncertainty [8]. The clean energy index may also serve as an efficient hedge and safe haven amid a crisis [9]. Moreover, EPU significantly influences GBI in the long run, with positive and negative effects across most quantiles, but opposite effects are only significant in higher quantiles [10]. However, Positive and negative shocks to GPR have an adverse short-term impact on GB returns. In the long term, GB returns are positively impacted by negative shocks in GPR and negatively affected by positive shocks in GPR [11]. Previous studies have limited literature on safe properties in various asset classes, with a focus on green assets during the 2008 financial crisis and the COVID-19 pandemic. This study examines the safe haven properties of seven assets, including ethical asset indices and a conventional stock index, during geopolitical crises, economic policy uncertainty, and stock market volatility. It allows investors to match their portfolios with their values and gives stakeholders the knowledge they need to make wise decisions.

Despite having profound growth, literature on the safe haven status of the ethical asset class is very inadequate. This study contributes to this gap from three dimensions. Firstly, we consider two types of ethical financial assets: environmental or green ethical assets (SPESG, MSCIESGL, SPGRNB, SPCLN, DJSI) and religious ethical assets (e.g., Islamic asset class; DJIM). We also add a conventional asset (SPG1200) to make a comparison. Secondly, the impact of uncertainty is time- and context-varying. We have considered three types of uncertainties: 1) the geopolitical risk (hereafter, GPR) index; 2) the economic policy uncertainty (EPU) index; and 3) CBOE market volatility (VIX) index. Thirdly, conventional analytical methods often fail to find the hidden dependencies and correlations in asset prices [12]. Hence, we have employed both novel Quantile coherence and Wavelet coherence. to capture the time-varying relation and various market states (bearish, normal, and bullish), respectively, because the literature suggests that the impact of uncertainties on assets varies depending on time and market conditions. Also, we employ extreme shocks from uncertainty factors analysis. Furthermore, we decompose our data into three frequencies, short, medium, and long, to comprehend the time-frequency, time domain and lead-lag associations between risks and seven asset classes, which would help investors to make better forecasting decisions.

Alongside this, the timeframe considered in this study (2011–2021) covers some important global, regional, and economic risk events, including the most recent COVID-19 pandemic. There are several rationales for selecting these assets. First, these assets have been established in the past literature as safe-haven assets, particularly against conventional asset classes, e.g., stocks, precious metals, bonds, etc. Second, these assets have shown a strong potential to withhold uncertainty shocks. The study assumes the potential of these asset classes for investors seeking to build a sustainable portfolio for a low-carbon future.

Our findings suggest that all seven assets can provide a safe haven against geopolitical risk (GPR) due to the positive or neutral impact of GPR shocks in all markets, frequency (excluding bullish markets with monthly frequency), and time domain. Most observed coherencies between economic policy uncertainty (EPU) and the seven assets are positive at lower quantile levels during monthly frequencies and middle to upper quantile levels during weekly frequencies. These results indicate that the selected assets can help hedge EPU shocks in a range of market regimes. On the other hand, during economic crises and COVID-19 pandemics, most assets, except the S&P Green Bond (SPGRNB) and S&P Global Clean Energy (SPCE) indices, are negatively impacted by EPU. Furthermore, all asset index returns, except SPGRNB, are particularly vulnerable to the movement of the volatility index (VIX). However, in the yearly and monthly frequencies, high stock market volatility (bullish market) has a positive influence on the MSCI World ESG Leaders (MSCIESGL) and SPGRNB. These results indicate their safe-haven capabilities against high stock market volatility regimes.

Clearly, the investors and the fund managers can redesign their portfolios and investment strategies based on these findings. Alternative investment opportunities, demand for safe-haven assets, and a growing climate agenda make these findings important for policymakers as well. While we assume that investors may prefer to switch to some of the green bonds and climate-friendly assets, it is important to recognize the limited availability of these ethical asset classes. Keeping the supply constraints constant, by evaluating both ethical and conventional stock indices against unforeseen vicissitudes in GPR and EPU shocks, portfolio risk managers and investors can balance their capital allocation strategies to win over several risks and market conditions. An ideal portfolio involving green investments will help hedge against GPR and EPU. It is worth noting that ethical assets do not help construct efficient portfolios due to the uncertainties arising from the VIX, particularly in the normal market. We hope that environmentally conscious investors and asset managers act as the vanguard of a new investment standard by combining diverse ethical asset categories in different quantiles and frequencies to diversify the risk and safeguard the value. Moreover, ethical investment research advocates for sustainable, equitable, and responsible finance and business practices. It empowers stakeholders to make well-informed decisions that benefit society and enables investors to align their portfolios with their values, thereby promoting responsible consumption. Companies that prioritize ethics encounter fewer regulatory issues, scandals, and public backlash, ultimately leading to more stable long-term returns. Policymakers have the opportunity to create incentives for ethical investment, thus enhancing market stability and societal welfare.

We discuss the literature in section two, followed by methodologies in section three, and results in section four. Lastly, section five provides the concluding remarks of this study.

## 2 Review of related literature

Ethical assets represent an emerging sector within the financial markets. These instruments have gained attraction as they allow investors to support various sustainable initiatives. This also helps them diversify their portfolios and align financial goals with environmental values. Outperformance is highlighted by studies such as those by Reboredo, Ugolini [13] & Tang, Zhang [14], which indicate that sustainable investments can deliver robust financial returns. However, the pricing of assets presents a complex challenge, as it is influenced by a myriad of factors beyond just environmental impact. Macroeconomic considerations, including geopolitical risks, economic policy uncertainty, and volatility index, play a significant role in the valuation and return of ethics. As such, investors and issuers must navigate these risks to maintain profitability and market stability. Broadstock, Cheng [15] underscore the importance of managing these risks effectively, suggesting that a comprehensive approach to the interplay between environmental and societal factors is crucial.

### 2.1 Global uncertainty and risk factors

Over the last ten years, several significant risk factors have impacted global financial markets and economic progress. The war in Ukraine has been the most recent event that has affected the global food supply, energy prices, and the average prices of everyday commodities [16]. A depressing economic growth in the post-COVID-19 pandemic period that was directly impacted by the prolonged uncertainty and destruction of the global value chain has left us with new lows every day in the financial markets. Investors' confidence was negatively affected due to the prolonged uncertainty associated with these adverse events [2]. Other recent key crises include the likes of Russia's annexation of Crimea, the Arab Spring, the global refugee crisis, and Brexit. Such upheavals amplified political and economic instability and global uncertainty while the world continues to advance quickly [17].

The GPR and EPU indexes track specific terms in newspaper coverage to measure geopolitical risk and economic policy uncertainty. They primarily use North American newspapers but have global significance. The GPR and EPU indexes exhibit noticeable spikes during certain events, such as the Russian invasion of Ukraine and the COVID-19 pandemic [18]. The literature on volatility transmission is crucial for modern risk management. The volatility associated with the COVID-19 crisis is significant for both risk and asset management. It is essential to assess whether the risks posed by this crisis differ from those of previous crises. The VIX has emerged as the primary indicator of risk volatility in the financial sector [19]. Climate change and environmental degradation are considered global risk events. The profound impact that poor quality of the environment has on every aspect of human life is being emphasized by the governments and financial market authorities. Consequently, we have witnessed several key changes to how corporations can consume natural resources to achieve net zero and other sustainable growth agendas. For instance, the United Kingdom and China are set to achieve net zero initiatives by the year2050^3^ and 2060, respectively. The sustainability agenda has set forth dos and don'ts that are part of the investor screening for choosing a new investment [20,21]. Since climate factors are global risk factors, investors generally have a positive perception of the stability and performance of these assets in the financial markets. Therefore, ethical assets are generally considered safe.

Geopolitical risks (GPR) are vital factors in investment decisions that impact (stock) market volatility and carry significant economic consequences [22]. The high geopolitical risk reduces trading activity, lowers stock returns, and causes capital plight off the developing economies into the economies that enjoy less uncertainty. Baker [23] stipulate that innovations in EPU anticipate decreases in investment, production, and employment. High volatility in the stock market and a lower drive for investment are sensitive to uncertainty in policy. EPU has been the focal point of a growing strand of literature [24,25]. These studies generally focus on the impact of uncertainties on economic outputs and asset prices.

Market-based uncertainty arises from financial market volatility, which is generally measured using the VIX index introduced by the Chicago Board Options Exchange (CBOE). The VIX is a substitute for corporate stock market risks and a measure of market uncertainty that fits best with developed markets and industries. The VIX index helps understand market liquidity and depth, which help shape investment decisions [17]. Alongside, volatility indexes also incorporate information on future volatility and are used as a proxy for indicating market sentiment. Literature using VIX is on the rise that looks into how market volatility influences market trading and economic outputs [4,26,27].

### 2.2 Ethical and safe-haven assets

Environment, Social, and Governance (ESG) assets have experienced considerable performance improvements in recent decades. Corporations report their ESG engagement not only because of regulatory pressure but also to protect their reputation among competitors and consumers. Investors and consumers take ESG as the ‘right thing to do’ that helps preserve the global climate. Therefore, assets engaging with ESG or being categorized as ESG assets generally experienced lower perceived risk [[28], [29], [30]]. On similar grounds, these assets also help diversify investment risk. Innovations in line with sustainability gave birth to a new breed of financial assets, e.g., GBs and clean energy. As the market demand grows, countries find it crucial to introduce green bond legal frameworks that incentivize issuers (borrowers) with fiscal benefits owing to their contributions to global environmental issues [31]. Preference for a safer world motivates investors to buy and hold on to these bonds while the borrowers are pledged to invest the proceeds in projects that uphold environmental protection and sustainable climate safety. Consequently, 1) investors go beyond a simple risk-return matrix while buying these assets, 2) they rely on their belief and values-system to invest in these ventures, and 3) they replace other risky assets with these ethical assets at times of crisis.

Higher demand pays the bill. A stiff demand shift for these ethical stocks, both among retail as well as institutional investors, means that investors are ready to pay a large amount to buy GBs [7]. Literature has seen a surge in academic works targeting several areas of ethical asset performance, such as the ESG [[32], [33], [34]], MSCI ESG Leaders (MSCIESGL) [13,30] GB [8,35], clean energy [10,36,37], and the Dow Jones Sustainability Index [38,39].

Most studies that cover ethical stocks emphasize ESG and the COVID-19 pandemic period, among other risk factors. Rubbaniy et al. [40] find that due to strong positive co-movement between the ESG and COVID-19 fear indices, ethical stocks carry strong safe haven and hedging properties over a medium-term (32–64 days frequency). However, the same attributes do not hold against market volatility (taking VIX as a proxy), particularly in the short-term frequency (0–8 days). Using data from the Arab region for a duration that covers COVID-19, Mousa [41] find that amid the post-pandemic phase, the volatility of ESG stocks was lesser than a composite market index for relatively medium to long terms as the shocks of the pandemic affected the ESG stocks only for a shorter period.

Piserà, Chiappini [42] scrutinized the safe haven and hedging status of the ESG assets from the Chinese market during COVID-19. While they reported strong risk hedging properties of the ESG stocks, they did not have a safe-haven attribute. The study also reported ESG stocks having higher hedging benefits over cryptocurrency. While analyzing the safe haven between ESG stocks and precious metals, Lei, Xue [43] found palladium and gold to be short-period safe havens against ESG markets in North America, Europe, and the developed Asia-Pacific. During the COVID-19 outbreak, the results were robust.

Islamic stocks are also considered ethical because of their strict ethical screening criteria. The Dow Jones Islamic Market World (DJIM) is being used in several studies as the proxy for Islamic ethical assets to investigate the impact of diverse types of shocks, including the GPR, EPU, and OVX [24,44,45]. However, the results are mixed. Arif et al. [46] examined the safe haven and diversification benefits of Islamic equities during the pandemic and global financial meltdown. The study found that Islamic equities in the G7 markets lacked safe haven features generally but had strong, safe haven properties during the COVID-19 epidemic. Delle Fogile, Panetta [47] conducted a systematic literature review of the safe properties of Islamic and conventional stocks. They, however, did not report any significant difference between the two competing sectors on the safe haven properties.

Hasan et al. [25] explored the safe haven potential of precious metals, cryptocurrencies, and Islamic equities amid the pandemic and worldwide economic downturn in 2008. They revealed that Islamic stocks were the safe havens during the pandemic as well as the global financial crisis. However, gold and bitcoin were stronger safe haven assets during severe market recessions. Islamic stocks were found to be safe haven assets investments during the economic downturn exhibited by Ref. [48]. Hassan et al. [49] however, found that Gulf Cooperation Country Islamic stock indices failed to safeguard investors throughout the pandemic and the 2008 worldwide financial crisis; sovereign bonds exhibited the most diversification benefit.

### 2.3 Ethical assets and uncertainty factors

Recent research has delved into the impact of geopolitical threats on the green economy, examining factors such as institutions, internal conflicts, religion, economic and social issues, and military power [11]. Mauerhofer [50] proposes that sustainable investment is influenced by the effective implementation of sustainability regulations, closely tied to upholding law and order. Additionally, Caldara, Iacoviell [51] have found that geopolitical risks significantly shape investment decisions. Furthermore, Tian et al. [52] have explored the differential effects of geopolitical risks on the green bond markets of China, Europe, and the United States over both short and long terms. Positive and negative shocks to GPR have an adverse short-term impact on GB returns. In the long term, GB returns are positively impacted by negative shocks in GPR and negatively affected by positive shocks in GPR [11]. Ha [53] presents evidence of connectivity between geopolitical risk, renewable energy volatility, and the green bond market during the health pandemic and the period of the Russia invasion of Ukraine. The interplay between economic policy uncertainty (EPU), geopolitical risk (GPR), and the transition to clean energy is a multifaceted subject with significant implications for global financial markets and sustainable development. Recent studies, such as those by Liu et al. [54] have delved into these dynamics, exploring how shifts in energy policy can align with Sustainable Development Goals (SDGs) and mitigate climate change. Consequently, the market for green energy has experienced a notable growth in demand, which may lead to increased stock prices for green energy entities. As Zhao et al. [55] note, the price of renewable energy also responds to the consequences of uncertainty during periods of crisis, such as the COVID-19 epidemic and the Ukrainian war crisis.

Other research has also explored the impacts of economic policy uncertainty (EPU) and geopolitical risk (GPR) indexes on global financial markets. Furthermore, studies have recommended transitioning from fossil energy to clean energy to address climate change issues and meet Sustainable Development Goals (SDGs) [54]. As a result, there has been a notable surge in demand for the green energy market, potentially leading to increased stock prices for green energy companies. Additionally, the prices of renewable energy also respond to uncertainty during crisis periods, such as the COVID-19 pandemic and the Ukrainian war crisis [55,56]. Moreover, Ha demonstrates the link between geopolitical risk, renewable energy volatility, and the green bond market during the health pandemic and the Russia invasion of Ukraine [53].

Previously, researchers have looked into how well financial assets like cryptocurrency, commodities, and international stocks can protect against inflation, economic policy uncertainty (EPU), global economic policy uncertainty, and the VIX (volatility index). However, there has been little research on the hedging and safe haven properties of environmentally friendly (green) assets against different uncertainties. Examining the connection between green securities and macroeconomic variables could open up a new area of research [19].

In summary, past studies reported safe haven features in diverse asset classes, but rarely on green assets and more often on the comparison of the two major shocks: the global financial crisis in 2008 and the COVID-19 pandemic in early 2020. Literature on safe properties and various global uncertainties is limited at best. Moreover, the analysis techniques used in various extant studies are limited in understanding the frequency, complexity, and dependence of uncertainty and asset prices. This study fills these gaps by inspecting the safe haven possessions of seven assets – six ethical asset indices and one conventional stock index – amid geopolitical crises, economic policy uncertainty, and volatility in the stock market. Quantifying the dependency structure between the two time-series variables is critical for analyzing cross-correlation under various market conditions and investment horizons. So, we used two sophisticated methodologies: Quantile Coherence and Wavelet coherence, by taking into consideration multiple market circumstances (bearish, normal, and bullish) at various frequency scales (short-, medium-, and long-term) and the lead-lag connection.

## 3 Data and empirical methodology

### 3.1 Data and summary statistics

This study examines the ethical (SPESG, MSCIESGL, SPGRNB, SPCLN, DJSI, and DJIM) and non-ethical conventional (SP1200) asset performance against three types of uncertainties (GPR, EPU, and VIX). We extract the daily closing price data from October 3, 2011 to September 30, 2021. Refer to Table 1 for details of the data.

**Table 1:** Definition and data sources of selected variables.

Dimensions	Variables	Notations	Data Sources
**Uncertainty indices**	Geopolitical risk	GPR	www.matteoiacoviello.com
	Economic policy uncertainty	EPU	www.policyuncertainty.com
	The CBOE Volatility Index	VIX	www.investing.com
**Asset classes**	S&P Global 1200 ESG	SPESG	www.spglobal.com
	S&P Green Bond	SPGRNB	
	S&P Global Clean Energy	SPCE	
	Dow Jones Sustainability World	DJSI	
	Dow Jones Islamic Market World	DJIM	
	S&P Global 1200	SPG1200	
	MSCI World ESG Leaders	MSCIESGL	www.msci.com

Table 2 displays essential descriptive data for the daily returns series of assets studied in this study. The results suggest that mean returns are lower and almost identical in most asset classes. The highest and lowest volatilities are observed in the case of clean energy (SPCE) and green bond (SPGRNB), respectively. However, regarding uncertainty, GPR exhibits greater volatility, followed by EPU shocks.

**Table 2:** Descriptive analysis and unit root tests.

Variables	Mean	SD	Skew	Kurt	Jarque-Bera	ADF	PP
SPESG	0.03	0.69	−0.78	30.29	113628.9∗∗∗	−38.25∗∗∗	−55.55∗∗∗
MASCIESGL	0.03	0.68	−0.75	31.98	128037.4∗∗∗	−38.12∗∗∗	−55.79∗∗∗
SPGRNB	0.01	0.28	−0.15	15.76	24770.11∗∗∗	−39.10∗∗∗	−58.23∗∗∗
SPCE	0.02	1.09	−0.29	17.90	33816.58∗∗∗	−36.75∗∗∗	−60.61∗∗∗
DJSI	0.02	0.73	−0.83	26.44	83953.87∗∗∗	−38.22∗∗∗	−55.64∗∗∗
DJIM	0.03	0.74	−1.06	28.00	95740.04∗∗∗	−40.56∗∗∗	−60.61∗∗∗
SPG1200	0.03	0.69	−0.76	28.99	103098.8∗∗∗	−38.25∗∗∗	−55.35∗∗∗
GPR	0.00	53.13	0.15	4.92	573.00∗∗∗	−55.16∗∗∗	−256.36∗∗∗
EPU	0.00	51.81	−0.01	6.64	2012.11∗∗∗	−37.50∗∗∗	−237.88∗∗∗
VIX	−0.02	5.98	0.78	11.39	11074.84∗∗∗	−59.01∗∗∗	−61.06∗∗∗

Notes: The table reports the summery statistics. SD stands for standard deviation, and Skew and Kurt are used to denote skewness and kurtosis. The ADF and PP are the Augmented Dickey-Fuller and Phillips-Perron test statistics for unit roots that take into account constants and trends. The ‘∗∗∗’ signify the significance level at 1 %.

To validate the use of the quantile-based technique, we use the BDS (Broock, Dechert, and Scheinkman) test to assess the non-linearity of our series. Table 3 shows the BDS test results based on time series residuals using a vector autoregressive (VAR) technique in many dimensions (m = 2, 3, 4, …, 6). The null hypothesis of linearity is rejected for all the cases; thus, the non-linearity is further evidenced when implanting diverse dimensions in the BDS test. Consequently, all the diagnostic tests stress us choosing a non-linear estimate technique, which can capture the heterogeneous association between the time series.

**Table 3:** BDS test results for nonlinearity from the VAR model-based residuals.

Variables	m = 2	m = 3	m = 4	m = 5	m = 6
SPESG	0.03∗∗∗	0.05∗∗∗	0.06∗∗∗	0.06∗∗∗	0.06∗∗∗
MSCIESGL	0.03∗∗∗	0.05∗∗∗	0.06∗∗∗	0.06∗∗∗	0.06∗∗∗
SPGRNB	0.02∗∗∗	0.03∗∗∗	0.03∗∗∗	0.02∗∗∗	0.02∗∗∗
SPCE	0.03∗∗∗	0.05∗∗∗	0.05∗∗∗	0.05∗∗∗	0.04∗∗∗
DJSI	0.03∗∗∗	0.04∗∗∗	0.05∗∗∗	0.05∗∗∗	0.05∗∗∗
DJIM	0.01∗∗∗	0.03∗∗∗	0.04∗∗∗	0.04∗∗∗	0.05∗∗∗
SPG1200	0.03∗∗∗	0.05∗∗∗	0.06∗∗∗	0.06∗∗∗	0.06∗∗∗
GPR	0.02∗∗∗	0.04∗∗∗	0.04∗∗∗	0.04∗∗∗	0.04∗∗∗
EPU	0.02∗∗∗	0.04∗∗∗	0.04∗∗∗	0.05∗∗∗	0.04∗∗∗
VIX	0.03∗∗∗	0.05∗∗∗	0.05∗∗∗	0.05∗∗∗	0.04∗∗∗

Notes: The table presents the BDS test results, which assesses the null hypothesis of independence and identical distribution (i.i.d.) for the time series against an unspecified alternative hypothesis. ∗∗∗ represent the significance level at 1 %.

**Fig. 1:**
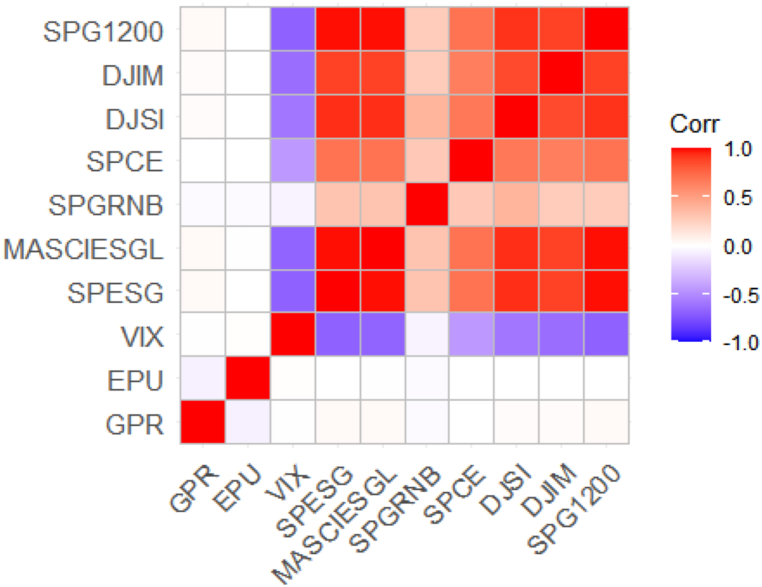
Visualization of the correlation matrix.

Fig. 1 visualizes the correlation matrix. The correlation matrix demonstrates that the GPR and EPU have a zero or near zero correlation with all the asset classes, highlighting the potential of hedging or safe-haven ability. However, VIX has a high negative connection with all asset classes (except SPGRNB). This finding implies that the asset classes chosen in this study are adversely impacted by market volatility.

### 3.2 Quantile coherency model

We utilize the dependence structure between numerous ethical and non-ethical asset classes and several uncertainties, especially their quantile connectedness in the joint tails and frequencies of distribution. To capture this, we utilize a quantile cross-spectral dependence methodology (QS) that was originally developed by Kley, Baruník [57]. In a range of market conditions (bearish, normal, and bullish markets), as well as over multiple investment periods (i.e., short, medium, and long-term), this technique can recognize a factor's connection with another, thus, can help in identifying the assets' hedge and safe-haven traits against diverse uncertainties more appropriately.

Like previous research, we define a hedge as an asset's returns having positive coherence (no or low coherence) with changes in uncertainty on average, indicating a robust (weak) hedge against uncertainties. A strong (weak) safe haven exists when an asset's returns show positive coherence (no or low coherence) with changes in uncertainty under extreme shocks.

Following conventions stated in extant literature [[57], [58], [59]], this study sets two stringent stationary processes of factors ${\left(R_{t}\right)}_{t\epsilon z}$, with mechanisms $R_{t}=\left(R_{t,x,}R_{t,y}\right)$, where x and y are substitutions for a couple of variables. Hence, in the following way (Equation (1)), the quantile coherency between these two procedures $\left(R^{x,y}\right)$ can be outlined as follows:

(1) $$R^{x,y}\left(\omega ;\tau_{1},\tau_{2}\right)≔\frac{f^{x,y}\left(\omega ;\tau_{1},\tau_{2}\right)}{{\left(f^{x,x}\left(\omega ;\tau_{1},\tau_{1}\right)f^{y,y}\left(\omega ;\tau_{2},\tau_{2}\right)\right)}^{1/2}}\text{,}$$

where −π<ω<π implies the quantile cross-spectral density and $\left(\tau_{1},\tau_{2}\right)∊\left[0,1\right]$. $f^{x,y}$, $f^{x,x}$, and $f^{y,y}$ state to the quantile spectral densities of procedures $R_{t,x,}$ and $R_{t,y}$, which can be achieved using the Fourier transform of a matrix of quantile cross-covariance kernels $\Gamma \left(\tau_{1},\tau_{2}\right)≔{\left(f\left(\omega ;\tau_{1},\tau_{2}\right)\right)}_{x,y}$, where (Equation (2))

(2) $$\Upsilon_{k}^{x,y}≔Cov\left(I\left\{J_{t+k,x}\leq q_{x\left(\tau_{1}\right)}\right\},I\left\{J_{t,y}\leq q_{y\left(\tau_{2}\right)}\right\}\right)\text{,}$$

for x, y∊{1,…,d}, k∊z, $\tau_{1},\tau_{2}∊\left[0,1\right]$, and scenario A is specified by function I{A}. We obtain an urgent indication of the cross-section dependency and serial correlation, respectively, by selecting x ≠ y and diversifying k. The quantile cross-spectral density kernel matrix is produced by this frequency's domain as $f\left(\omega ;\tau_{1},\tau_{2}\right)≔{\left(f\left(\omega ;\tau_{1},\tau_{2}\right)\right)}_{x,y}$, where

(3) $$f^{x,y}\left(\omega ;\tau_{1},\tau_{2}\right)≔{\left(2\pi \right)}^{-1}\;\sum_{k=-\infty }^{\infty }\Upsilon_{k}^{x,y}{\left(\tau_{1},\tau_{2}\right)e}^{-ik\omega }$$

We estimate quantile coherency as follows (Equation (4)), based on Baruník, Kley [57] utilizing smoothed quantile cross-periodograms:

(4) $${\hat{G}}_{n,R}^{x,y}\left(\omega ;\tau_{1},\tau_{2}\right)≔\frac{2\pi }{n}\;\sum_{s=1}^{n-1}W_{n}\left(\omega -\frac{2\pi s}{n}\right)I_{n,R}^{x,y}\left(\frac{2\pi s}{n},\tau_{1},\tau_{2}\right)\text{,}$$

Where $I_{n,R}^{x,y}$ and $W_{n}$ denote rank-based copula cross-periodograms matrix and order of weight functions respectively. Then, in the following way (Equation (5)), the quantile coherency valuation can be expressed:

(5) $${\hat{R}}_{n,R}^{x,y}\left(\omega ;\tau_{1},\tau_{2}\right)≔\frac{{\hat{G}}_{n,R}^{x,y}\left(\omega ;\tau_{1},\tau_{2}\right)}{{\left({\hat{G}}_{n,R}^{x,x}\left(\omega ;\tau_{1},\tau_{1}\right){\hat{G}}_{n,R}^{y,y}\left(\omega ;\tau_{2},\tau_{2}\right)\right)}^{1/2}}$$

This research examines the coherency matrices in connection with three quantiles labeling to upper (0.95), average (0.5), and lower (0.05) quantiles, with the amalgamation of three quantile levels—right (0.95|0.95), middle (0.5|0.5), and left (0.05|0.05)—tails of the joint distribution. Additionally, we separate the coherency into three time horizons: quarterly high value, half-yearly medium value, and annual low value corresponding to ω∊2π{1/3, 1/6, 1/12}.

From Equation (3), we can decompose the cross-spectral density of kernels {$f^{x,y}\left(\omega ;\tau_{1},\tau_{2}\right)$} into real and imaginary parts [57]. The actual portion denotes the co-spectrum of the processes ${\left(I\left\{R_{t,x}\leq q_{x}\left(\tau_{1}\right)\right\}\right)}_{t\epsilon z}$ and ${\left(I\left\{R_{t,y}\leq q_{y}\left(\tau_{2}\right)\right\}\right)}_{t\epsilon z}$,^4^ while the imaginary portion represents the quadrature spectrum, which removes a number of sources of noise coherence. Nonetheless, we only provide the actual portions of our quantile coherency calculations due to readability and improved display considerations.^5^

### 3.3 The wavelet coherence (WC)

As a robustness test, we further analyze our data using the wavelet coherence (WC) approach to explore the lead/lag association between asset classes and uncertainties. It decomposes data into time-frequency space, capturing short-term and long-term patterns. This helps understand market behaviors and interactions between asset classes. The lead-lag relationship is crucial for portfolio management and risk assessment. Wavelet coherency and phase relation techniques reveal correlation and directional intensity over time, providing insights into causality and connectedness. This wavelet coherence (WC) approach eventually proves that the findings of previous methodologies are quite robust and reliable. The WC method, developed by Whitcher, Craigmile [60], helps scrutinize the time-varying and intensity of co-dependence between pairs in time and frequency domains. Additionally, any possible non-stationarity, nonlinearities, structural breaks, or seasonal patterns in a pair's connection may be controlled with this method [58]. We make use of the cross-wavelet transform (WT) in conjunction with the WC [[59], [60], [61]]. Among the couple of series x(t) and y(t), here is an illustration of the cross-wavelet using continuous wavelet transformations:

(6) $$W_{x,y}\left(u,s\right)=W_{x}\left(u,s\right)W_{y}^{*}\left(u,s\right)\text{,}$$

where Equation (6) is the cross-wavelet transformation, and s and u are the scale and position index, respectively. The wavelet transforms of x(t) and (t) are signified by $W_{x}\left(u,s\right)$ and $W_{y}\left(u,s\right)$, respectively, where $\prime \prime {*}^{\prime \prime }$ denotes the complex conjugate. According to Torrence and Webster [62], the co-movement between the two series, *x(t)* and *y(t)*, across time and frequency may be evaluated by the WC and is expressed as follows (Equation (7)):

(7) $$R^{2}\left(u,s\right)=\frac{|S\left(s^{-1}W_{xy}\left(u,s\right)\right){|}^{2}}{S\left(s^{-1}|W_{x}\left(u,s\right){|}^{2}S|W_{y}\left(u,s\right){|}^{2}\right)}\text{,}$$

where, S states the smoothing operator between time and frequency, recognized by $S\left(W\right)=s_{scale}\left(s_{time}\left(W_{n}\left(s\right)\right)\right)$. The coefficient of $R^{2}\left(u,s\right)$ justifies the conditions $0\leq R^{2}\left(u,s\right)\leq 1$ in the time-frequency space. $R^{2}\left(u,s\right)$ values closer to zero indicate a poor correlation between the two-time series, which are shown as blue, while values closer to one indicate a high connection, which are shown as warmer (red) colors. There is no link between the series in the cold (blue) zones. Since the WC's theoretical distribution is unknown in this instance, the statistical significance of the coherence is investigated at the 5 % significance level using Monte Carlo simulations [62]. Nevertheless, it is impossible to discern between the lead-lag connection and positive or negative coherency due to the squared value of WC. Torrence and Compo, present a wavelet phase-difference analysis to capture these insufficiencies [62].

(8) $${Ø}_{xy}\left(u,s\right)=\tan^{-1}\left(\frac{J\left(S\left(s^{-1}W_{xy}\left(u,s\right)\right)\right)}{R\left(S\left(s^{-1}W_{xy}\left(u,s\right)\right)\right)}\right)\text{,}$$

where (Equation (8)), the WC phase differences are stated by ${Ø}_{xy}\left(u,s\right)$. The unreal and real parts of the smoothed power spectrum are denoted by J and R, respectively, and are shown with arrows inside the regions classified as having high coherence. The two directions shown by the black arrows are the lead/lag phase relationship and the negative or positive correlation between the couples. The WC estimations across asset classes and uncertainty indices are shown in Fig. 4, Fig. 6. The vertical axis' monthly frequencies, such as 4, 8, and 16, represent the short-, medium-, and long-term investment horizons, respectively.^6^

### 3.4 Extreme shocks from uncertainty factors

This section looks at how our sample assets have performed against the ten largest shocks that stemmed from three uncertainty factors during the sample period. Table 4 illustrate the returns of SPG1200, DJIM, SPESG, DJSI, SPGRNB, SPCLEN and MSCIESGL in the ten largest daily shocks of uncertainty indicators. We notice that the COVID-19 pandemic was responsible for most of the largest shocks to uncertainty indices, as these shocks happened in 2020 when COVID-19 struck the world unprecedentedly. However, Panel A of Table 4 reports the returns of SPG1200, DJIM, SPESG, DJSI, SPGRNB, SPCLEN and MSCIESGL on the ten biggest shocks derived from the GPR index. The results show that SPG1200, SPESG, DJSI and MSCIESGL returns are positive for six of the ten days; DJIM shows positive returns throughout the whole 10 days, and both SPGRNB and SPCLEN exhibit positive returns for four out of ten days.

**Table 4:** Ten major shocks from five uncertainty factors.

Panel A: Ten biggest shocks from GPR								
Date	GPR	SPG1200	DJIM	SPESG	DJSI	SPGRNB	SPCLEN	MSCIESGL
07-01-2020	420.29	−0.090	0.013	−0.123	−0.195	−0.225	−0.144	−0.116
09-01-2020	402.58	0.646	0.863	0.579	0.411	−0.155	0.553	0.585
17-11-2015	361.02	−0.050	0.001	−0.061	−0.037	−0.126	−0.220	−0.055
18-11-2015	318.07	−0.050	0.001	−0.061	−0.037	−0.126	−0.221	−0.055
16-11-2015	297.27	0.390	0.258	0.480	0.409	−0.047	−0.928	0.439
12-04-2018	292.84	0.447	0.395	0.396	0.355	−0.365	−0.008	0.407
09-05-2018	289.47	0.636	0.639	0.663	0.447	0.022	−0.528	0.555
05-06-2017	274.77	−0.065	0.027	−0.062	−0.066	−0.035	−0.094	−0.063
06-08-2019	272.20	0.513	0.745	0.518	0.189	0.091	0.646	0.547
19-09-2016	266.86	0.148	0.289	0.141	0.279	0.038	0.482	0.150

**Panel B: Ten biggest shocks from EPU**								

17-05-2020	861.10	1.006	0.004	0.911	0.753	0.053	1.538	0.945
26-04-2020	855.17	0.553	0.008	0.565	0.482	0.036	0.737	0.599
05-05-2020	807.66	0.959	1.244	1.012	1.208	−0.215	1.142	1.043
29-03-2020	743.13	0.702	0.001	0.774	0.863	−0.022	−0.098	0.959
05-04-2020	739.55	1.861	0.001	1.859	1.603	−0.020	1.864	1.934
02-04-2020	738.02	1.290	1.503	1.380	0.896	0.454	0.907	1.333
26-12-2020	699.78	0.076	0.267	0.071	0.033	0.097	−0.265	0.045
26-03-2020	670.30	4.612	4.683	4.725	4.768	2.013	3.274	4.934
22-03-2020	651.07	−1.020	−0.004	−1.018	−1.122	0.035	−2.038	−1.091
27-04-2020	642.66	0.550	1.427	0.562	0.480	0.036	0.732	0.595

**Panel C: Ten biggest shocks from VIX**								

16-03-2020	82.69	−3.345	−9.632	−3.333	−2.986	−0.180	−3.575	−3.426
18-03-2020	76.45	−5.212	−4.415	−5.061	−4.482	−2.410	−10.558	−5.227
17-03-2020	75.91	3.993	3.871	3.992	3.666	−1.587	6.101	4.029
12-03-2020	75.47	−9.976	−9.303	−10.220	−10.61	−2.367	−12.497	−10.250
15-03-2020	74.40	−3.237	−0.003	−3.225	−2.90	−0.180	−3.452	−3.312
19-03-2020	72.00	−0.267	0.493	0.554	0.775	−1.298	1.371	0.751
14-03-2020	66.12	−3.135	−0.003	−3.125	−2.818	−0.180	−3.337	−3.206
20-03-2020	66.04	−2.327	−2.229	−2.365	−1.335	−0.375	1.372	−2.717
27-03-2020	65.54	−2.487	−2.396	−2.509	−2.346	0.763	−5.140	−2.487
21-03-2020	64.56	−1.010	−0.004	−1.008	−1.109	0.035	−1.998	−1.080

**Note:** The table shows the returns of all assets against the ten biggest uncertainty indices shocks.

Additionally, Panel B reveals the returns of all seven assets on ten extreme shocks in EPU and demonstrates that both SPGRNB and SPCLEN have positive returns on seven out of ten days; Moreover, positive returns for other all remaining assets are documented throughout the ten largest daily shocks. Furthermore, Table 4 Panel C demonstrates that out of the ten biggest shocks, all assets (except DJIM) have eight positive returns in the VIX index. Conversely, SPG1200 return is negative in the biggest shock days in the oil markets, indicating that these assets are likely to be negatively influenced by oil market volatility. According to the analysis above, SPG1200 return seems to be more volatile in the extreme shocks of the sample uncertainty indicators compared to all other assets.

## 4 Results and discussions

### 4.1 Quantile coherency (QC) estimations

Based on Taylor (2008), the returns of the chosen set can be distinguished by positively significant autocorrelations as a constant evolution of return volatility throughout time. To reduce the largest source of autocorrelations, we employ the GARCH (1, 1) model^7^ proposed by Barunk, Kley [57], which can normalize the return series by volatility.

**Fig. 2:**
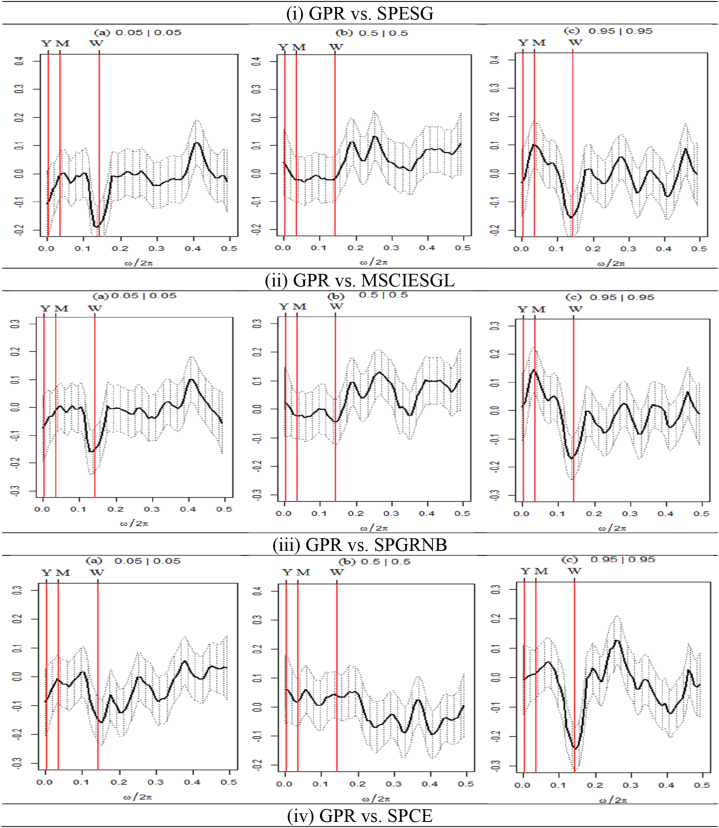

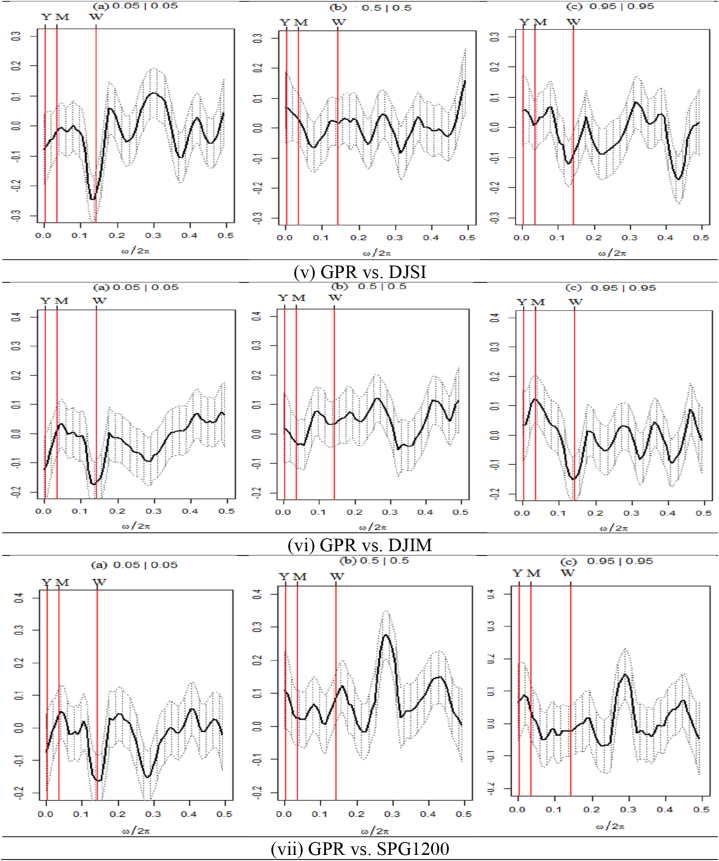

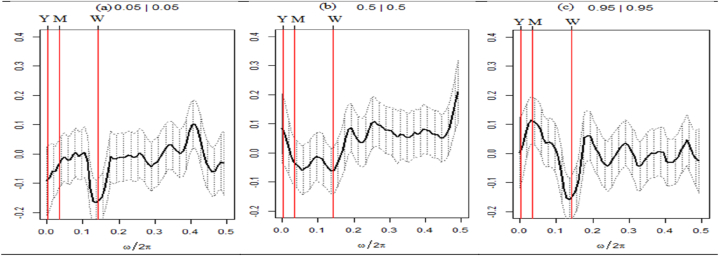
Quantile coherence plots between GPR and seven assets. Notes: The figure depicts the quantile coherence of GPR and seven assets. Each panel describes the relationship between GPR and seven selected assets, e.g., SPESG (i), MSCIESGL (ii), SPGRNB (iii), SPCE (iv), DJSI (v), DJIM (vi), and SPG1200 (vii). The real component of quantile coherency is presented for 0.05, 0.5, and 0.95 quantiles at a 5 % significance level, as per Baruník and Kley (2019). The horizontal axis shows the monthly cycle (0–0.5), denoted by ω/2π. The vertical axis reflects the measure of codependence between a pair of variables. W, M, and Y represent weekly, monthly, and annual time periods, respectively.

**Fig. 3:**
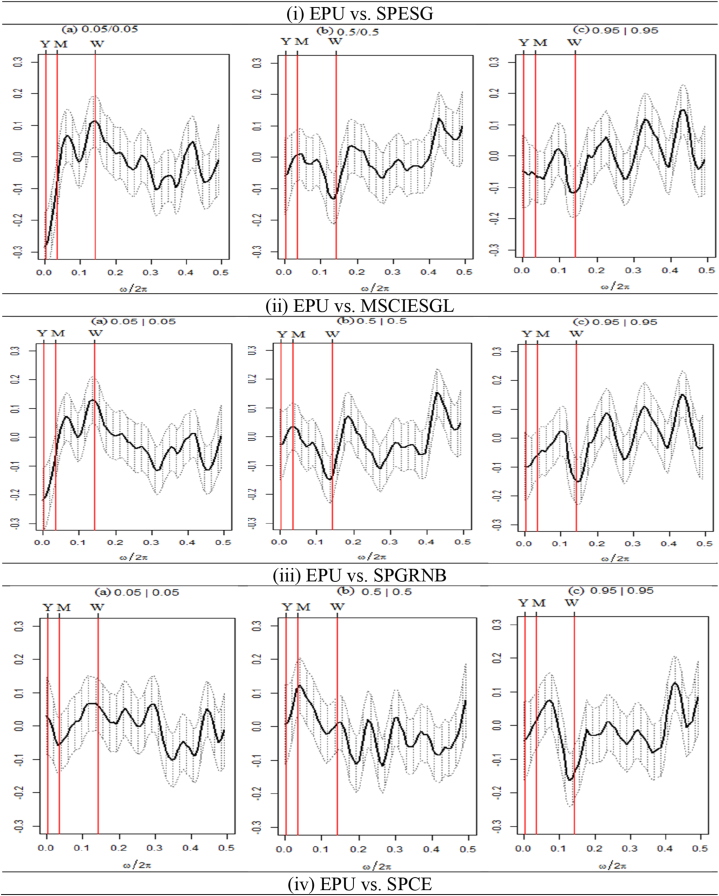

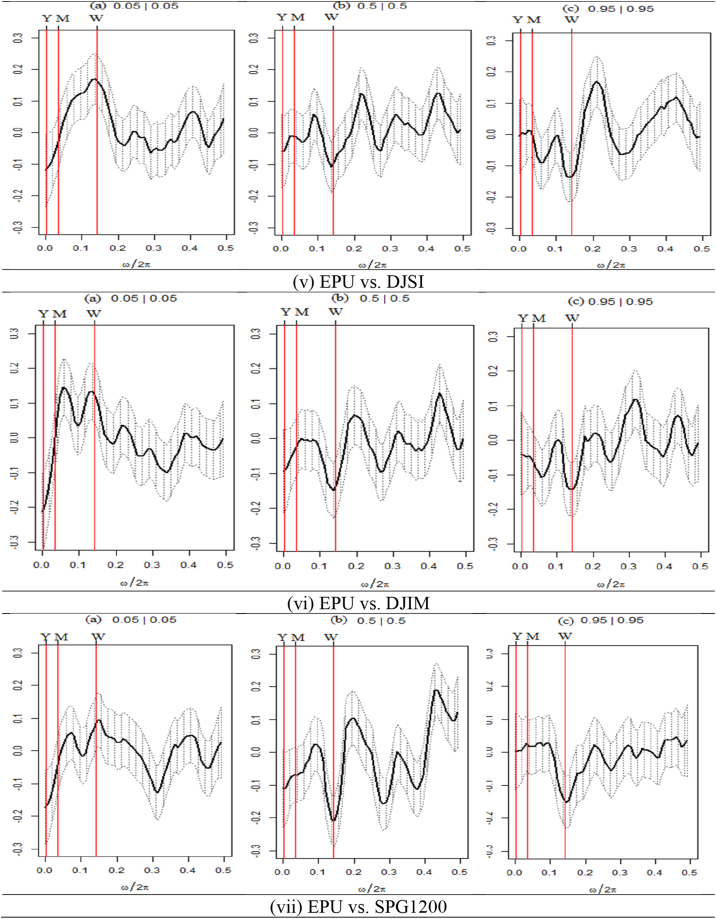

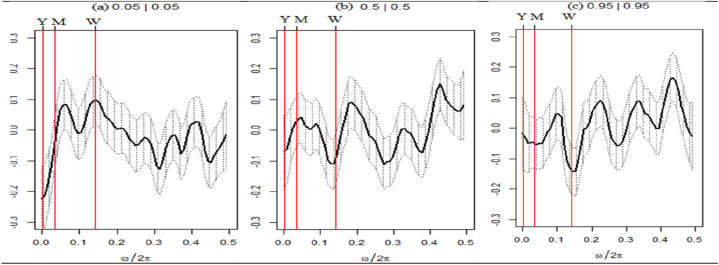
Quantile coherence plots between EPU and seven assets. Note: Each panel describes the relationship between EPU and seven selected assets, e.g., SPESG (i), MSCIESGL (ii), SPGRNB (iii), SPCE (iv), DJSI (v), DJIM (vi), and SPG1200 (vii). The real component of quantile coherency is presented for 0.05, 0.5, and 0.95 quantiles at a 5 % significance level, as per Baruník and Kley (2019). The horizontal axis shows the monthly cycle (0–0.5), denoted by ω/2π. The vertical axis reflects the measure of codependence between a pair of variables. W, M, and Y represent weekly, monthly, and annual time periods, respectively.

**Fig. 4:**
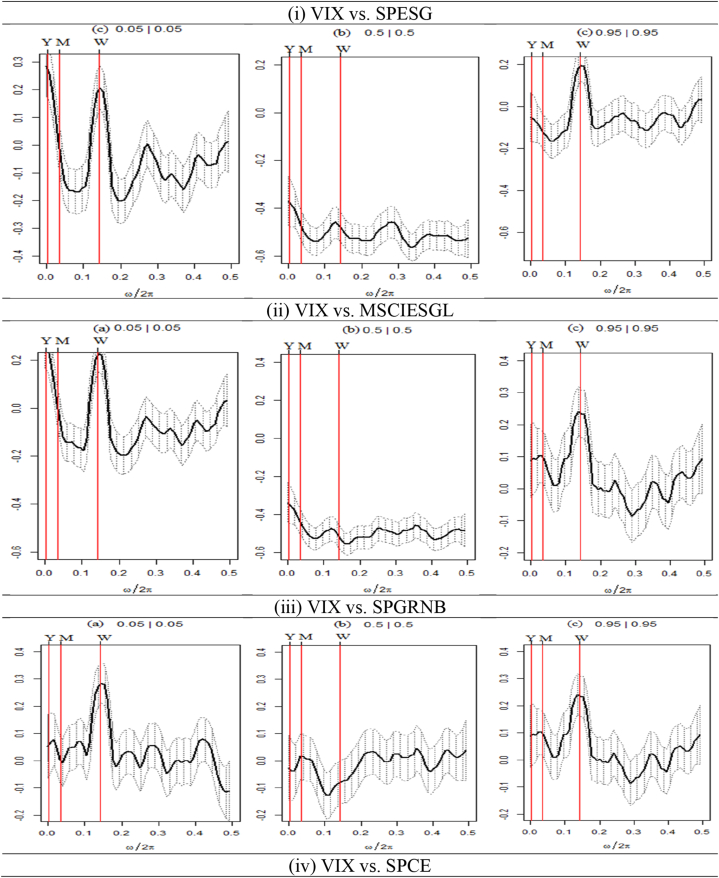

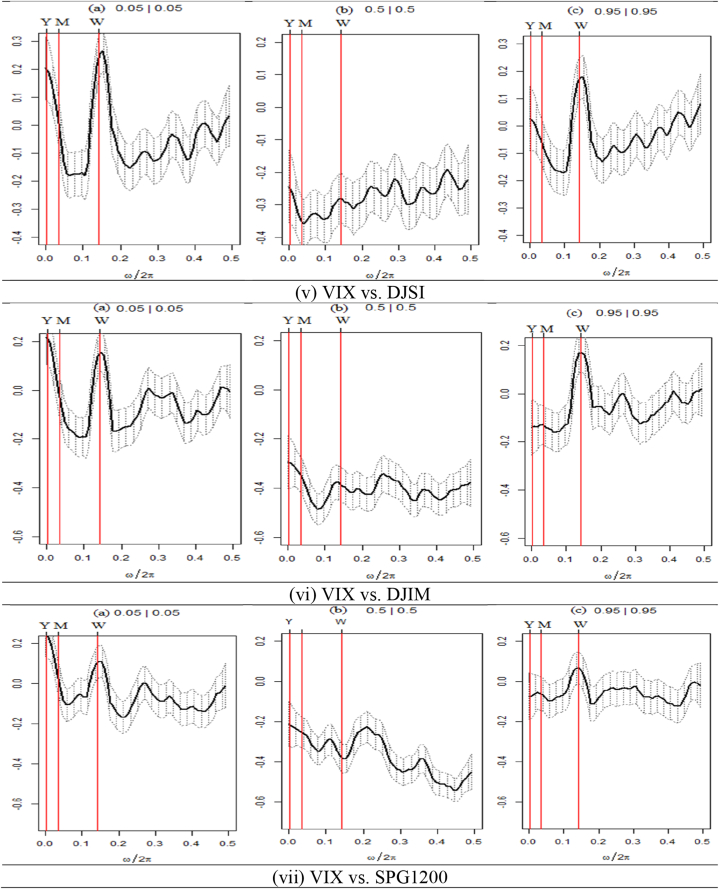

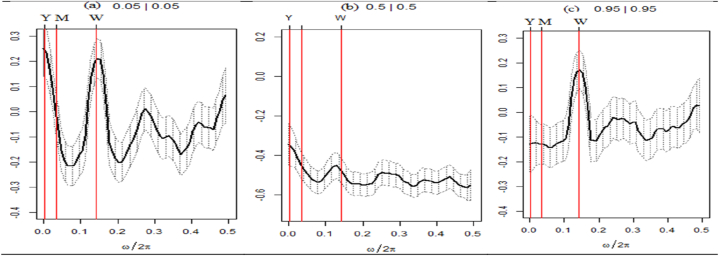
Quantile coherence plots between VIX and seven assets. Note: Each panel describes the relationship between VIX and seven selected assets, e.g., SPESG (i), MSCIESGL (ii), SPGRNB (iii), SPCE (iv), DJSI (v), DJIM (vi), and SPG1200 (vii). The real component of quantile coherency is presented for 0.05, 0.5, and 0.95 quantiles at a 5 % significance level, as per Baruník and Kley (2019). The horizontal axis shows the monthly cycle (0–0.5), denoted by ω/2π. The vertical axis reflects the measure of codependence between a pair of variables. W, M, and Y represent weekly, monthly, and annual time periods, respectively.

We present the outcomes of the quantile coherence estimations in Fig. 2, Fig. 3, Fig. 4. The figures show the higher (0.95|0.95), middle (0.05|0.05), and lower (0.05|0.05) quantiles. On the horizontal axis, the co-dependence of two series is displayed together with the daily cycle over the interval information.

Fig. 2 depicts the quantile coherency estimations of GPR paired with every asset considered in this study. Fig. 2(i) displays an average negative coherence for the GPR-SPESG pair across the lower return quantile (0.05|0.05). Conversely, a significant positive co-movement between GPR and SPESG is noticed in terms of short-run (weekly) frequency in normal return quantile (0.5|0.5), while both medium- and long-term horizon reveals a negative coherence. However, a positive upward movement is observed for the long-term horizon, though the correlation experiences a downward trend after crossing the yearly horizon in the upper return quantile (0.95|0.95).

Similar patterns of coherence are clear in the case of the GPR-MSCIESGL pair (Fig. 2(ii)), where a significant positive correlation exists in the average quantile for short-term and upper quantile for long-term frequencies. Consequently, against GPR, SPESG has strong hedging properties only in the normal market condition in short-term frequency, while MSCIESGL contains strong, safe haven opportunities in long-term frequency. Ahad et al. [63] also suggest that a safe haven portfolio with a one-month investment horizon can yield positive returns during wartime, suggesting investors should consider ESG indices as safe assets during crises.

The quantile correlation between GPR and SPGRNB, shown in Fig. 2(iii), demonstrates an average negative association in most quantile and frequency scales. However, in normal market conditions, both long- and medium-term returns show a significant positive co-movement. In bearish market conditions, only long-term coherence is significantly positive, while a non-continuous peak is observed in the weekly horizon in the upper quantile. On the other hand, in Fig. 2(iv), the GPR-SPCE pair exhibits negative dependence in both yearly and monthly frequencies, whereas the weekly frequency unveils an average positive association in the lower quantile.

Furthermore, the normal return quantile reveals that SPCE has a significant favorable connection with GPR in the yearly horizon and an average negative association in the monthly horizon. An average positive or zero correlation in the weekly investment horizon is noticed. However, in the upper return quantile, a significant positive coherence exists in the long-term until the mid of the medium-term horizon, while an average negative association is concluded in short-term frequency. These findings imply that SPGRB offers hedging opportunities in the long-term, whereas the SPCE provides the same both medium- and long-term frequencies for normal market conditions. Both SPGRB and SPCE provide a strong safe haven in long-term frequency only in bullish market conditions.

Fig. 2(v) illustrates an average positive dependence structure between GPR and DJSI in normal return quantile, except for the long-term. Conversely, a strong positive upward coherence in the long-term quantile is noticed, though it turns to a negative region after crossing the middle of the monthly horizon. For the rest of the time and frequency scales, even in the case of extremely lower quantiles, the coherence between GPR and DJSI is negative on average. These results entail the weak hedging capabilities of DJSI against GPR for medium- and short-term returns in normal market conditions. DJSI can provide strong, safe haven benefits during the buying pressure market condition. Sustainable development objectives, according to Oliveira et al. [64], can assist asset managers, investors, and other stakeholders in reducing the detrimental consequences of local and global measures of uncertainty.

As depicted in Fig. 2(vi), it can be observed that the GPR-DJIM pair exhibits a steadily adverse link with the extremely lower return quantile across various time-frequency intervals. On the other hand, it can be observed that the GPR-DJIM pair displays a significant positive coherence over the time span in the quantile associated with normal returns. This suggests that the pair has considerable potential for hedging in typical market conditions. Notwithstanding the robust positive long-term coherence, bull market conditions exhibit a negative association on average in both the short- and medium-term periods. The findings suggest that DJIM has the potential to serve as a hedging instrument, particularly in typical market circumstances across various frequencies. Additionally, DJIM can function as a robust hedge and a safe haven against GPR shocks in the long-term frequency when the market is bullish. This discovery is in line with the research conducted by Hasan et al. [61], which posits that the conservative nature of Islamic markets, characterised by limitations on interest, gambling, and speculative practices, renders them less vulnerable to financial upheavals.

Fig. 2(vii) demonstrates a positive coherence between GPR and SP1200 in the weekly timeframe in the lower return quantile. Additionally, a robust positive correlation is evident in the short-term period in the normal quantile. Furthermore, it has been observed that a favorable negative correlation exists in the annual timeframe for the normal return quantile. Conversely, a favorable positive correlation is evident for the higher quantile in the GPR-SP1200 pair. However, in all other instances, there exists a feeble unfavorable correlation between them.

Fig. 3 displays the quantile coherency of EPU paired with all the assets. Fig. 3(i) exhibits an average negative connection between EPU and SPESG in the lower quantile at the weekly frequency and a strong negative association in the yearly frequency, whereas a strong positive association is found in the monthly frequency. Conversely, in the case of normal and upper return quantiles, we notice a negative association in the long- and medium-term frequencies, while a positive coherency is observed in the short-term frequency. Furthermore, the MSCIESGL-SPESG pair in Fig. 3(ii) exhibits similar findings. These findings imply that both MSCIESGL and SPESG may have hedging potentials against EPU in the medium-term frequency at bearish market conditions. The hedging opportunity is weak in normal and bullish market conditions for short-term frequency.

Turning to the EPU-SPGRNB pair, in lower quantiles, Fig. 3(iii) reports an average positive association between EPU and SPGRNB at medium- and short-term frequencies. Conversely, in the extremely higher return quantile, the pair shows an average negative association except for monthly frequency. Except for the short-term frequency in normal return quantile, there is a favorable co-movement in long- and medium-term frequency. Hence, based on these results, SPGRNB can be used as a weak hedging tool. These results are similar to Pham, Nguyen's [8] that GBs and uncertainty are weakly related during periods of relatively lower uncertainty, allowing GBs to be utilized as a hedge against EPU.

Fig. 3(iv) demonstrates a favorable association between EPU and SPCE for the monthly frequency at lower quantiles, while it turns negative in the weekly horizon. Conversely, the EPU-SPCE pair in normal return quantile reveals an average positive coherence in both short and medium-term frequencies and a strong positive co-movement in higher return quantiles, except for medium-term. Thus, our findings suggest that SPCE can hedge the EPU strongly in medium-term frequency at bearish market conditions while serving as weak hedging for the normal return quantile. However, except for medium-term frequency, SPCE contains both hedge and safe-haven potentials at bullish market conditions against EPU.

Regarding Fig. 3(v), it can be observed that only the medium-term horizon within the lower quantiles manifests a robust positive correlation between DJSI and EPU. A typical positive relationship is observed in the context of short-term frequency during a market condition characterized by a bullish trend. Fig. 3(vi) demonstrates a consistent positive coherence in the medium-term frequency at lower quantiles and an average association in the short-term horizon for DJIM. An observation can be made regarding the positive coherence of DJIM, which is found to be consistent across various investment horizons. Fig. 3(vii) demonstrates that the EPU-SPG1200 pair displays exclusively robust positive interdependence during medium-term frequency in a bearish market environment.

In contrast, it is observed that normal and bullish market conditions exhibit a tendency towards positive returns on average in the short term. The aforementioned findings demonstrate that SPCE, DJIM, and SPG1200 assets can serve as effective hedging instruments for medium-term investors seeking to mitigate the adverse effects of EPU in bearish market conditions. Conversely, in bullish market conditions, investors may solely benefit from hedging and safe-haven strategies to mitigate exposure to economic policy uncertainty (EPU).

The quantile dependence structure between seven selected assets paired with VIX is depicted in Fig. 4(i)–(ii),(iii),(iv),(v),(vi)&(vii). Most pairs demonstrate a highly unfavorable co-movement, except for certain frequencies and quantiles. Initially, the pairs demonstrate that at lower return quantiles, the long-term horizon displays a robust positive correlation with VIX. However, a maximum positive coherence is observed at the intersection of the weekly and monthly horizons for equally extreme market conditions. In contrast, each asset set exhibits a highly negative correlation with the VIX under typical market circumstances. However, SPGRNB (as depicted in Fig. 4(iii)) displays a moderately positive correlation on average across short-term market conditions. The VIX-SPGRNB pair exhibits a robust positive correlation in both monthly and yearly timeframes, even during periods of heightened market volatility. Thus, drawing from these results, it can be concluded that only SPGRNB exhibits robust safe-haven characteristics at both medium- and long-term frequencies when compared to VIX. The findings indicate that all seven assets exhibit robust hedging characteristics in the long term during bearish market scenarios. However, it is noteworthy that only SPGRNB demonstrates a feeble hedging capability in the short term compared to VIX across all market conditions.

### 4.2 Wavelet coherence analysis

**Fig. 5:**
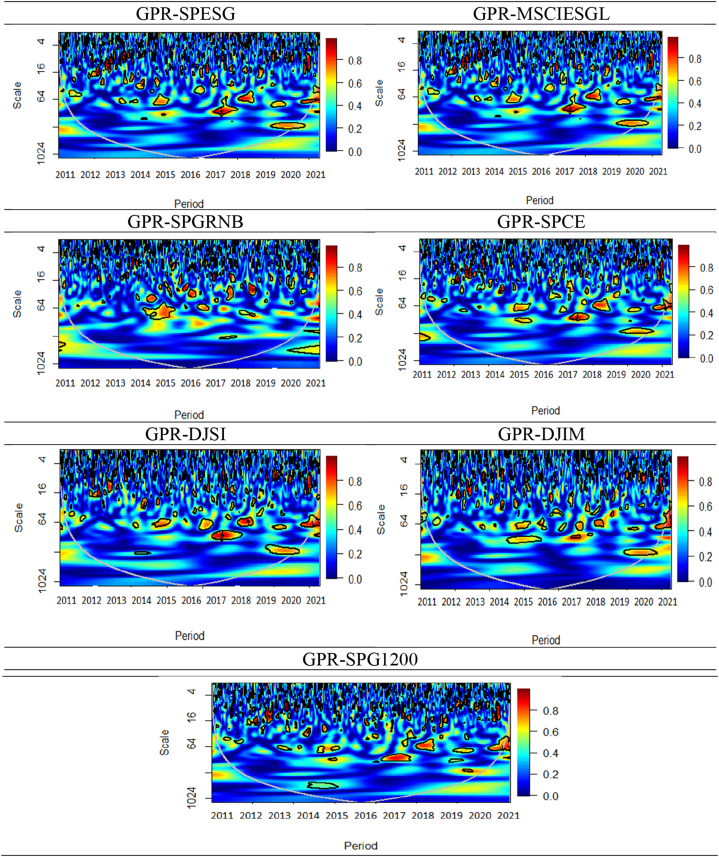
Wavelet coherence between GPR and seven assets. **Note:** The figures exhibit the wavelet coherence (WC) pairwise plots between GPR and seven assets. The horizontal axis displays time frames, whereas the vertical axis represents frequency in days. The arrows show the phases that correlate to the variables. The two series are in phase (anti-phase), as shown by the arrows on the right (left) side. The first (second) variable is shown as leading by the arrows, which are pointing right-side down (up) and left-side up (down).

**Fig. 6:**
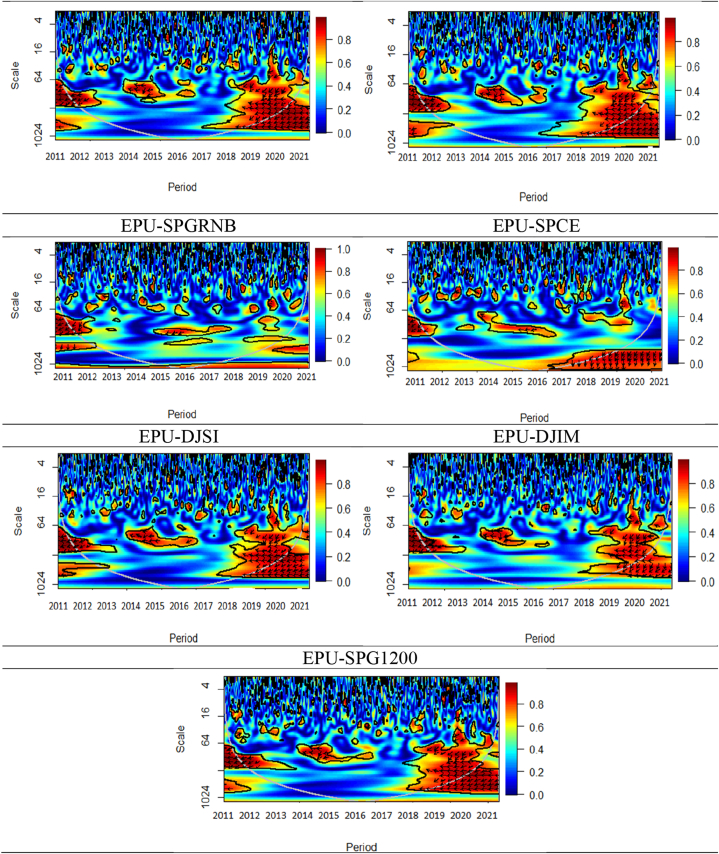
Wavelet coherence plots between EPU and seven assets. Note: The figures exhibit the wavelet coherence (WC) pairwise plots between EPU and seven assets. The horizontal axis displays time frames, whereas the vertical axis represents frequency in days. The arrows show the phases that correlate to the variables. The two series are in phase (anti-phase), as shown by the arrows on the right (left) side. The first (second) variable is shown as leading by the arrows, which are pointing right-side down (up) and left-side up (down).

**Fig. 7:**
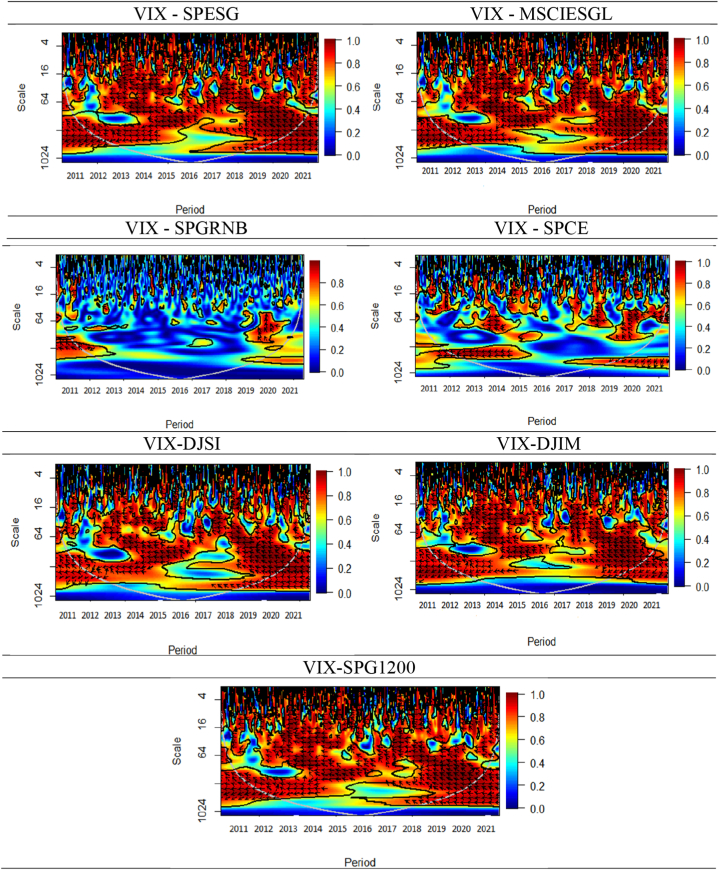
Wavelet coherence plots between VIX and seven assets. **Note:** The figures exhibit the wavelet coherence (WC) pairwise plots between VIX and seven assets. The horizontal axis displays time frames, whereas the vertical axis represents frequency in days. The arrows show the phases that correlate to the variables. The two series are in phase (anti-phase), as shown by the arrows on the right (left) side. The first (second) variable is shown as leading by the arrows, which are pointing right-side down (up) and left-side up (down).

To further provide comprehension into the co-movement of variables under study in the time-frequency domain, the WC methodology is employed. Fig. 5, Fig. 6, Fig. 7 depict the relationships between three global risk and uncertainty factors and seven distinct asset classes in terms of their WC plots. WC metric evaluates the time-frequency associations as well as the causal relationships between the respective variable pairs. The time dimension is represented by the horizontal axis, while the frequency dimension is depicted by the vertical axis. The degree of association is shown by the color on the right side of the bar chart. From blue (near low coherence) to red (high coherence), the coherence color code is used. The black U-shaped curve represents the cone of influence (COL). The areas of significant coherence within COL are delineated by dark contours. The scale (number of days) shows the frequency domain, highlighting that the short, medium, and long-term frequency domains imply the short, medium, and long-run investment horizons, respectively.

The graphical representation depicted in Fig. 5 illustrates the WC plot of a set of seven assets in conjunction with the GPR. It is observed that within the COL, the time-frequency planes are predominantly characterized by cool hues for each respective index. This

suggests a lack of coherence between all assets and GPR. All indices appear to be devoid of any influence from GPR and offer a secure refuge against GPR shocks, even amidst the COVID-19 pandemic.

The pairwise WC plot between the EPU and seven assets is also presented in Fig. 6. The seven assets are depicted in a cold color, suggesting limited connectivity to the EPU across various timeframes and magnitudes. Despite this, several small but significant islands within the COL exhibit a red coloration accompanied by leftward-pointing arrows, indicating an anti-phase relationship within the medium-to-long-term frequency range of 64–1024 days. This pattern was observed during 2012, 2014–2015, and 2019–2021, except for the SPGRNB-EPU plot. This suggests that while assets generally offer a secure investment option, they may exhibit negative behavior with respect to EPU during times of economic downturn, stock market declines, and the COVID-19 pandemic, except for SPGRNB. The present study's results concur with those of Arif et al. [46] who established that the returns on GBs appear to have become more persistent following the emergence of the COVID-19 pandemic.

The outcomes of the analysis of the relationship between the VIX and seven financial assets, namely, SPESG, MSCIESGL, SPGRNB, SPCE, DJIS, DJIM, and SPG1200) are illustrated in Fig. 7. An anti-cyclic (out-of-phase) association is observed between VIX and many asset classes, except SPGRNB and SPCE, across all periods and time frames. Similarly, the long-term horizon of CDS-stock (CDS-VIX) pairs shows a strong negative (positive) association [65]. The directional movement of all arrows is towards the left, indicating a primarily leading effect by VIX. The findings indicate a substantial adverse connection between SPESG and the VIX, implying that SPESG is not able to effectively mitigate the risks associated with stock market fluctuations. Furthermore, it is observed that there exists a lack of coherence or absence of coherence between SPGRNB and VIX across all time and frequency domains. This leads to the conclusion that SPGRNB is not negatively impacted by VIX shocks. A mixed relationship between the SPCE and VIX is witnessed. In the short, medium, and long-term scales, certain arrows indicate a leftward (anti-phase) upward trend, implying a negative correlation between VIX and SPCE, with VIX leading. Notably, most islands in both the time and frequency domains exhibit a prevalence of cool hues, signifying a lack of coherence between SPCE and VIX.

### 4.3 Summary of the findings

The present study employs two distinct methodologies, namely quantile coherence and wavelet coherence, to comprehend the time-frequency, time domain and lead-lag associations between risks and seven asset classes. The quantile coherence findings suggest that the variables exhibit co-integration with time-frequency, indicating the existence of three distinct markets: bearish, normal, and bullish. In contrast, the wavelet coherence analysis reveals the presence of co-movement among the variables across various time domains, including short, medium, and long-run periods, along with quantile coherence.

Based on the quantile coherence analysis, we examine the relationship between various assets and the GPR and EPU. Our findings indicate that during a bullish market, all assets demonstrate safe haven properties against the GPR and EPU on a yearly and weekly basis, respectively. SPGRNB exhibits minimal correlations in this regard. Notably, the MSCIESGL and SPGRNB exhibit monthly and yearly frequencies, indicating their potential as safe-haven assets during elevated stock market volatility, as measured by the VIX. During a market downturn, the GPR has a negative impact on all assets, apart from SPGRNB. Conversely, EPU has a positive effect on assets at a monthly frequency. Doğan et al. [66] reveals the economic policy uncertainty indicator's impact varies across markets, emphasizing the significance of green bonds as a safe haven during uncertainty. Nonetheless, it is noteworthy that all assets function as hedges and safe-haven assets in relation to VIX on an annual basis while exhibiting a monthly correlation with SPGRNB. In a typical market scenario, except for DJIM exhibiting a positive response, all other assets demonstrate a neutral reaction towards GPR and EPU. However, across all frequencies, all assets exhibit a negative performance on the VIX, except for SPGRNB.

In addition to our quantile coherence, we have employed wavelet coherence analysis. It can be inferred that there is a minimal or negligible level of co-movement between GPR and the various assets studied. This suggests that these asset classes exhibit characteristics of safe-haven properties in relation to GPR. The EPU index has produced mixed outcomes, including negative co-movement and absence of co-movement. SPGRNB and SPCE are consistently regarded as safe havens, in contrast to other assets which exhibit negative associations during times of crisis such as economic growth recession (2011), stock market selloffs (2015), and the COVID-19 outbreak. Furthermore, regarding the VIX, it can be observed that all assets, except SPGRNB, experience a negative impact. Therefore, it is likely that SPGRNB and SPCE serve as secure assets that mitigate the potential risks and uncertainties associated with GPR, EPU, and VIX.

## 5 Conclusion and implications

In the next decade, corporations are expected to prioritize addressing climate change as a serious issue. Therefore, ethical assets have emerged as a viable investment option to protect investors from market downturns. This paper adds contributions to the existing knowledge on ethical finance, focusing on environmental and religious ethical aspects. Specifically, the study examines the quantile dependence of six ethical assets (SPESG, MSCIESGL, SPGRNB, SPCE, DJSI, and DJIM), as well as conventional assets (SPG1200), in relation to various risks and uncertainty indices (GPR, EPU, and VIX). The research utilizes two novel techniques, quantile coherence and wavelet coherence, during the time span of October 3, 2011, to September 30, 2021.

The outcomes suggest that GPR shocks have a favorable or neutral impact on all assets across different markets and time periods, except for bearish and bullish markets with monthly frequency. This implies that ethical assets can serve as a protective measure against GPR shocks. The study also investigates the potential hedging capabilities of environmental ethical (sustainability), religious ethical (DJIM), and non-ethical (SPG1200) assets against EPU. Most of the analyzed assets show favorable outcomes in terms of coherence with EPU at lower quantile levels in monthly frequencies and middle to upper quantile levels in weekly frequencies. This indicates that these assets have the potential to mitigate the impact of EPU shocks in various market states. However, except for SPGRNB and SPCE, the assets have been adversely affected by EPU shocks resulting from the COVID-19 outbreak and the economic crisis across diverse markets, frequencies, and time periods.

On the contrary, the returns of various asset indices exhibit significant susceptibility to fluctuations in VIX, indicating a lack of hedging capabilities against unexpected VIX disturbances. The positive trajectory that VIX exhibits under adverse market conditions can be advantageous to long-term investors. Conversely, the assets MSCIESGL and GRNB demonstrate a positive response to high stock market volatility, particularly during bullish market conditions, as evidenced by their performance in both yearly and monthly frequencies. This suggests that these assets possess a safe-haven characteristic that enables them to withstand periods of heightened stock market volatility. It is important to note that most assets have the ability to absorb risks associated with GPR, EPU, and VIX through diversification channels. Among the green assets, SPGRB and SPCE exhibit superior performance across all dimensions and offer opportunities for hedging and safe-haven investments.

The results of this study hold noteworthy ramifications for stakeholders such as investors, fund managers, policymakers, and governments in terms of sustainable development, improved investment strategies, and informed policymaking in diverse market conditions, investment timelines, and levels of uncertainty. By evaluating ethical and non-ethical indices in the face of unexpected fluctuations in GPR and EPU shocks, investors and risk managers can enhance their investment strategies to better navigate diverse risk and market conditions. An optimal portfolio can be constructed to diversify the risk of GPR and EPU. Furthermore, in cases where uncertainties arise from the Volatility Index (VIX), particularly in a typical market scenario, it is advisable for investors to refrain from investing in the assets that have been scrutinized in this research, as these assets are adversely impacted by fluctuations in the VIX. Nevertheless, investors and asset managers who prioritize environmental concerns may be leading the way toward a novel investment paradigm, and the financial sector plays a vital role in facilitating the transition toward an economy that aims to mitigate the impact of climate change. Beside this, investors who prioritize ecological considerations may opt to invest in environmentally friendly markets such as SPGRNB and SPCE, as they offer a secure investment option, portfolio diversification, and a commitment to sustainable practices.

Additionally, it is imperative for policymakers operating within these markets to remain cognizant of the unpredictable and hazardous scenarios that may arise as a result of market instability, remain abreast of evolving GPR and EPU shock circumstances and implement necessary measures to mitigate these risks. Upon assessing these factors, policymakers can effectively determine suitable courses of action to address potential risks and uncertainties. The present investigation adds to the extant body of literature by tackling significant environmental issues and furnishing persuasive proof that ethical markets, via their distinct risk-absorption mechanism, can proficiently absorb investment risk while mitigating and diversifying the risk of ambiguity.

While this study provides a solid foundation for future researchers, it is not exempt from limitations akin to other investigations. Our analysis is limited to a single conventional asset, assigned a specific level of risk and uncertainty. The integration of ethical assets with conventional assets, along with the analysis of various risk and uncertainty components, can facilitate a comprehensive understanding of the underlying interconnections among these factors. We have not also covered precious metals and cryptocurrency due to the potential complexity that may arise while finding a common coherence among these different types of markets.

### • Credit author statement

All authors equally contributed to the paper.

### • Availability of data and materials

The data sources are given in the data and methodology section of the paper. The datasets are provided on reasonable request.

### Funding

There are no funding sources.

## CRediT authorship contribution statement

**Md Bokhtiar Hasan:** Writing – review & editing, Writing – original draft, Supervision, Software, Methodology, Formal analysis, Conceptualization. **M. Kabir Hassan:** Writing – review & editing, Supervision, Conceptualization. **Mamunur Rashid:** Writing – review & editing. **Tanzila Akter:** Writing – original draft, Software, Methodology, Formal analysis, Data curation, Conceptualization. **Humaira Tahsin Rafia:** Writing – original draft, Software.

## Declaration of competing interest

The authors declare that they have no known competing financial interests or personal relationships that could have appeared to influence the work reported in this paper.

## Footnotes

1 https://www.bloomberg.com/professional/blog/esg-assets-may-hit-53-trillion-by-2025-a-third-of-global-aum/.

2 https://www.pwc.com/gx/en/news-room/press-releases/2022/awm-revolution-2022-report.html.

3 The Net Zero strategy of UK Government (https://www.gov.uk/government/publications/net-zero-strategy).

4 We estimate the co-spectrum by converting two-time series to Fourier form.

5 For the sake of conciseness, the plots are not displayed here, although the imaginary parts of the quantile coherency are also computed. Upon request, the findings are made accessible. Our results imply that the real and imaginary portions produce, for the most part, the same effects.

6 The scales following 16, such as 32 and 64, represent 32 and 64 months, respectively, indicating a longer investment horizon.

7 We examine it before executing the quantile coherence technique and find that the returns are not modified considerably; consequently, our results support the standardization of the return series. Results are accessible upon reasonable inquiry.
